# Caregiver burden and mental health among informal caregivers of older adults in Lebanon

**DOI:** 10.1186/s12982-026-01466-4

**Published:** 2026-01-31

**Authors:** Suzanne Charbaji, Khalil EI Asmar, Stephen J McCall, Ghada E Saad, Monique Chaaya

**Affiliations:** Department of Epidemiology and Population Health , American University of Beirut , Beirut, Lebanon

**Keywords:** Caregiver burden, Caregiver mental health, Lebanese older adults, Gender differences, Lebanon, Zarit-22, Self reporting questionnaire

## Abstract

**Background:**

The global population is aging, increasing demand for informal caregivers. In Lebanon – where caregiving is primarily family-based due to cultural norms and limited institutional care –research on caregiver burden and mental health is limited.

**Aims:**

This study provides the first community based, multi-governorate evidence from Lebanon quantifying caregiver burden, examining its association with mental health and exploring gender differences in an under-researched Arab sociocultural setting.

**Method:**

Data based on two cross-sectional studies (2013–2017) across three governorates. Among 739 informants of older adults, 144 informal caregivers providing occasional or frequent care were analyzed. Caregiver burden and mental health measured using validated tools (Zarit burden interview and self-reporting questionnaire). Bivariate and logistic regression analyses identified correlates and quantified the burden-mental health association.

**Results:**

Of the 144 caregivers, 10% (*n* = 14) reported caregiver burden and 19% (*n* = 27) experienced mental health disorders. Caregiver burden was strongly and directionally associated with worse mental health (OR = 17, 95% CI = 4–74; *p* < 0.001). Being female, living with care-recipient, providing hands-on care, and caring for someone who requires assistance most of the time significantly increased burden. All burdened caregivers were female.

**Conclusion:**

This community-based study provides new insight into caregiver burden and mental health in Lebanon. Caregiver burden in Lebanon appears low but may be under-reported due to cultural norms. When present, burden is strongly linked to poorer mental health, particularly among women. These findings should be interpreted as exploratory signals that align with global literature and highlight the need for culturally sensitive, gender-responsive caregiver support and mental health services. Results can inform policy and future research in Lebanon and similar Arab contexts.

**Supplementary Information:**

The online version contains supplementary material available at 10.1186/s12982-026-01466-4.

## Background

The global population is aging with older individuals nearly doubling from 9% in 2020 to 16% in 2050. The phenomenon, driven by increase in the life expectancy and a decrease in the birth rates [1], will increase the demand for the caregivers [2] as older people experience chronic health conditions, physical and cognitive decline [3]. In the Arab Region, family members, especially daughters, provide most elder care due to cultural values [4], lack of available alternative nursing homes, and weak elder-care policies including limited public funding, insufficient insurance coverage, and limited social or economic caregiver support [5].

Caregiver burden often results in physical and mental health ailments, financial struggles and social withdrawal [2], which can affect the quality of the caregiver’s lives and the quality of care and support they provide [6]. Several studies revealed consistent positive association between caregiver burden and caregiver mental health [7, 8] with female caregivers experiencing higher burden than males [7, 9–11]. Several theories, such as Wuest’s feminist caring theory and the Social constructivist theory, highlight the gendered nature of caregiving [9, 10]. Gerain and Zech’s “Informal Caregiving Integrative Model” integrates elements from the caregiver stress model and Job Demands-Resources model to have a more comprehensive understanding of caregiver experience and advocate for further research to improve caregiver support and quality of life [12].

Lebanon has the highest proportion of older adults among Arab countries [13] and this proportion is expected to increase to 21% by 2050 [14]. With strong family ties and very limited institutional care options, Lebanon relies heavily on informal caregiving, particularly by women.

Studies on caregiver burden in Lebanon are scarce. Existing evidence consists mainly of a conference poster and a preprint using a small convenience sample and focused on dementia caregivers in Lebanon rather than caregivers of older adults. Furthermore, these studies have not thoroughly explored the factors influencing caregiver burden [15, 16].

## Purpose of the study

This study aims to fill a critical evidence gap by assessing factors influencing caregiver burden and mental health among informal caregivers of older adults in Lebanon and exploring gender differences in caregiver experiences.

Guided by a modified “Informal Caregiving Integrative Model” [12], we hypothesize that caregivers who experience higher levels of burden have higher odds of mental health disorders, and that female caregivers experience a higher burden than male caregivers, potentially modifying the burden-mental health relationship.

## Methods

### Study design

This study is based on analysis of secondary data from two cross-sectional studies conducted between 2013 and 2017, in Lebanon, to assess dementia and other health issues of older adults aged 65 years and above. A multi-stage cluster sampling technique was employed to recruit a representative sample of community-dwelling older adults.

### Study population and sample

The first study collected data from 502 older adult participants from Beirut and two districts of Mount Lebanon governorate (Shouf and Aley) using a multi-stage random sampling approach [15]. Detailed sampling procedures are provided in Supplementary Appendix 1. The second study with a similar methodology took place in the Bekaa governorate where a representative sample of 237 was selected and interviewed [16]. Trained interviewers knocked on households to recruit participants and collect information using face to face interviews. Each older participant nominated an informant who they know very well and would report on the older participant’s cognitive health, on his or her own health, and caregiving when applicable. Informants who reported providing occasional or frequent assistance with daily tasks were classified as informal caregivers. Out of 739 informants, 144 met this criterion and will be used in the current analysis.

### Concepts and measures

Caregiver mental health, caregiver burden, caregiver’s socio demographic and socio economic factors in addition to caregiver setting are the variables used for the purpose of this study. The outcome of interest is caregiver mental health measured by the 20-item Self Reporting Questionnaire (SRQ). SRQ is a screening tool created by the World Health Organization for low and middle income countries to detect non psychotic mental health disorders such as depression, anxiety for past 30 days using a Yes/No answer format [17]. The SRQ score is the summation of the yes answers on the 20 dichotomous statements. The SRQ has been validated in many studies including its version in Arabic language [18]. The optimum cutoff point 5/6 showed a sensitivity of 82.35%, specificity of 84.46% and 84.29% correct classification [18]. Therefore, a total SRQ score of > = 5 indicated mental health disorders and was treated as binary variable (“no mental health disorders” vs. “mental health disorders”). The main exposure is caregiver burden measured by the 22 Long Zarit questionnaire, a 5 point Likert type scale. In 1980, Zarit et al. developed the 29 item scale to measure caregiver burden [19] and this instrument evolved with time into shorter versions. It is the most commonly used scale for measuring caregiver burden across diverse populations and it is a multi-dimensional construct with studies reporting between two and five factors [20]. It has shown good psychometric properties as measured by the scale’s validity and reliability and has been validated in several languages including Arabic [21]. The caregiver burden is computed as the summation of the answers to the 22 items of Zarit questionnaire with a score range between 0 and 88. Consistent with prior studies in the Eastern Mediterranean region using ZBA-I 22, the burden was dichotomized at a cutoff of 21 to indicate clinically meaningful caregiver burden. This cutoff has been commonly applied to reflect moderate burden in community-based and clinical caregiving studies allowing for meaningful comparison across similar populations [22]. Burden is treated as a binary variable (“no burden” vs. “burden”). The covariates were selected based on previous studies [7, 12, 23, 24] where socio demographic, socio economic and caregiving setting variables can be potential confounders whereas gender will be considered as an effect modifier [9, 10].

### Statistical analysis

Descriptive analysis summarized the distribution of caregiver burden, covariates (socio-demographic, socio-economic and caregiver setting) and caregiver mental health (frequency and proportions). Bivariate analysis using Yates-corrected Chi-square tested for significant correlates. Simple and Multiple Logistic regression quantified associations where co-variates that have a p-value < 0.05 at the simple binary logistic regression were selected to be included in the multiple binary logistic regression. The Hosmer-Lemeshow test assessed model fit. To examine effect modification by gender, we added a gender x burden interaction term (burden as continuous ZBI score). All statistical tests were conducted using level of significance α = 0.05. All statistical analyses were conducted using SPSS version 25. The proportion of missing data was low (< 5%). We performed a complete case-analysis and did not impute missing values.

## Results

### Descriptive analysis

The sample included 144 caregivers, with a mean age of 50 years (SD = 17). The majority of caregivers were females (111/144, 77%), married (84/144, 58%), not working (86/140, 61%), lived in Beqaa (80/144, 56%) and had completed intermediate or higher education (69/120, 58%). Most caregivers were immediate family caregivers (111/144, 77%), provided care occasionally (100/144, 69%), were co-residents (89/144, 62%) and served as the main hands-on caregivers (79/144, 55%). A small proportion of caregivers felt burdened (14/144, 10%). All caregivers who reported burden were women (14/14, 100%), compared to none among men. Among caregivers whose care recipients needed care most of the time, 12/44 (27%) reported burden. Mental health disorders were present in 27/144 (19%) of caregivers.

### Bivariate analysis

Table 1 presents the bivariate associations between caregiver burden, socio-demographic, socio-economic, and caregiving setting variables with mental health disorders. Caregiver burden (*p* < 0.001), caregiver education level (*p* = 0.029), and need for frequent care were significantly associated with the presence of mental health disorders. A significantly higher prevalence of mental health disorders was observed among caregivers with burden (11/14, 79%), low education (16/51, 31%) and those who reported that the care-recipient needed care much of the time (16/44, 36%). Gender, marital status, current work, location, co-resident, relationship to older adults, and type of care were not statistically significant predictors of mental health disorders.

**Table 1:** Distribution of main exposure(burden) and other factors (socio demographic, socio economic and caregiving setting) among sample of 144 caregivers and their bivariate association with outcome (mental health disorders)

Variables	Categories	Total *N* (%)	Mental health disorders *N* = 27 *N*(%)	*P*-value
Caregiver burden				
Caregiver burden	No	130(90)	16(12)	< 0.001*^a^
	Yes	14(10)	11(79)	
Caregiver socio-demographic & socio-economic factors				
Gender	Female	111(77)	21(20)	1.000
	Male	33(23)	6(18)	
Marital status	Not married	60(42)	12(20)	0.914
	Married	84(58)	15(18)	
Level of education	Low	51(42)	16(31)	0.029*
	Intermediate	38(32)	8(21)	
	High	31(26)	2(7)	
Current work	Not working	86(61)	18(21)	0.495
	Working	54(39)	8(15)	
Location	Beqaa	80(56)	20(25)	0.053
	Beirut/ML	64(44)	7(11)	
Caregiver setting				
Co-resident	No	55(38)	6(11)	0.094
	Yes	89(62)	21(24)	
Relationship to older adult	Not immediate family	33(22.9)	5(15)	0.727
	Immediate family	111(77.1)	22(20)	
Care arrangement	Need Care Occasionally	100(69.4)	11(11)	0.001*
	Need Care much of time	44(30.6)	16(36)	
Type of care	Organizational	65(45)	9(14)	0.249
	Main Hands-on	79(55)	18(23)	

*Indicates significant association between variable and outcome at *p* < 0.05

^a^ p-value by Fisher exact (1 cell expected count < 5)

All other p-values for 2*2 table are from Yates-corrected Chi-squared test (ꭓ^2^); p-value for 3*2 table is from Chi-squared test

Table 2 shows that gender (*p* = 0.04), co-residency (*p* = 0.026), care arrangement (*p* < 0.001), and type of care (*p* = 0.031) were significantly associated with caregiver burden. Burden occurred only among females (14/111, 13%), was more common among co-resident caregivers (13/89, 15%), those who felt that the care-recipient needed care most of the time (12/44, 27%) and hands-on caregivers (12/79, 15%). Marital status, level of education, current work, location, and relationship to older adults were not significant.

**Table 2:** Bivariate association with main exposure (caregiver burden) among sample of 144 caregivers

Variables	Categories	No burden *N* = 130(90%)	Burden *N* = 14 (10%)	*P*-value
Caregiver socio-demographic & socio-economic factors				
Gender	Female	97(87)	14(13)	0.0401*
	Male	33(100)	0(0)	
Marital status	Not married	53(88)	7(12)	0.704
	Married	77(92)	7(8)	
Level of education	Low	44(86)	7(14)	0.225
	Intermediate	32(84)	6(16)	
	High	30(97)	1(3)	
Current work	Not working	74(86)	12(14)	0.093
	Working	52(96)	2(4)	
Location	Beqaa	71(89)	9(11)	0.683
	Beirut/ML	59(92)	5(8)	
Caregiver setting				
Co-resident	No	54(98)	1(2)	0.026*
	Yes	76(85)	13(15)	
Relationship to older adult	Not immediate family	31(94)	2(6)	0.524^b^
	Immediate family	99(89)	12(11)	
Care arrangement	Need Care Occasionally	98(98)	2(2)	< 0.001^1^*
	Need Care much of time	32(73)	12(27)	
Type of care	Organizational	63(97)	2(3)	0.031*
	Main Hands-on	67(85)	12(15)	

*Indicates significant association between variable and outcome at *p* < 0.05

^b^ p-value by Fisher exact (1 cell expected count < 5)

All other p-values for 2*2 table are from Yates-corrected Chi-squared test (ꭓ^2^); p-value for 3*2 table is from Chi-squared test

Table 3 shows that the odds of having mental health disorders among caregivers who feel burden was 26 times that of caregivers who feel no burden (95% CI: 7–104). The wide confidence interval reflects the small number of burdened caregivers (*n* = 14) which may reduce the precision of the estimate. Having a high level of education and living in Beirut were associated with significantly lower odds of mental health disorders. Frequent care needs increased odds by 4.6 times (95% CI: 1.9–11). Other variables were not significantly associated.

**Table 3:** Unadjusted and adjusted odds ratios (OR) and their 95% confidence interval (CI) for different predictors of mental health disorders among a sample of 144 caregivers

Variables	Categories	Unadjusted OR(95% CI)	Adjusted OR model 2^c^(95% CI)
Caregiver burden			
Caregiver burden	No	1	-
	Yes	26(7–104)	17(4–74)
Socio-demographic and socio-economic factors			
Gender	Female	1	-
	Male	0.9(0.3–2.6)	-
Marital status	Not married	1	-
	Married	0.9(0.4–2.4)	-
Level of education	Low	1	-
	Intermediate	0.6(0.2–1.5)	-
	High	0.1(0.03–0.7)	-
Current work	Not working	1	-
	Working	0.6(0.3–1.6)	-
Location	Beqaa	1	-
	Beirut/ML	0.4(0.1–0.9)	-
Caregiver setting			
Co-resident	No	1	-
	Yes	2.5(0.9–6.7)	-
Relationship to older adult	Not immediate family	1	-
	Immediate family	1.4(0.5–4.5)	-
Care arrangement	Need Care Occasionally	1	-
	Need Care much of time	4.6(1.9–11)	2.2(0.8–6.3)
Type of care	Organizational	1	-
	Main Hands-on	1.8(0.8–4.4)	-

^c^Adjusted for care arrangement

### Multivariable analysis

All socio demographic, socio economic and caregiver setting variables that were significant at the bivariate level were entered individually along with caregiver burden into separate multivariable model to assess how they influenced the association between burden and mental health. When adjusted for education, location, and care arrangement separately, the odds ratio for caregiver burden decreased, with the greatest decrease observed when adjusting for care arrangement. Table 3 shows that, in the model including caregiver burden, care arrangement, and mental health, the adjusted OR for burden dropped to 17, and care arrangement was no longer statistically significant, suggesting that the initial unadjusted association between care arrangement and mental health may have been confounded by caregiver burden. Including additional variables such as education and location did not meaningfully change the adjusted odds ratio for caregiver burden beyond what was observed in the burden–care arrangement–mental health model. Therefore, we retained this parsimonious model for reporting, as it provided similar explanatory power without unnecessary complexity. The Hosmer-Lemeshow test results indicated a p-value of 0.698 indicating that the multiple logistic regression model had a good fit.

### Effect modification by gender

There was no statistical significance for the coefficient of the interaction term, indicating that gender was not an effect modifier.

## Discussion

Surprisingly, only 10% of caregivers in our sample felt burdened which is much lower than 30% to 70% reported in previous studies [25–27]. This discrepancy might be partly explained by the profile of our care recipients, as most caregivers reported providing care occasionally (69% of sample), suggesting lower care needs. For caregivers whose care recipients needed care most of the time, the reported burden of 27% is in line with prior Lebanese findings [26]. Another reason for this discrepancy is the cultural norms in which caregiving may be perceived as a familial duty [4], potentially leading to underreporting of burden driven by social desirability or reporting biases. In such contexts, caregivers, particularly women, may hesitate to report burden, resulting in more conservative burden estimates and weaker observed associations. Regional comparisons further support this interpretation. For example, our overall reported burden appears similar to findings from a cross-sectional study of 315 informal caregivers of elderly patients in Saudi Arabia, where the majority of caregivers (73%) reported no to moderate burden [28]. Likewise, a recent Jordanian study among dementia caregivers reported mild to moderate burden level with burden increasing alongside disease severity and care demands [29]. These regional findings support the notion that cultural expectations of familial caregiving potentially contributes to underreporting of burden. Nevertheless, our finding of 27% burden among caregivers of high need older adults aligns with regional findings from dementia-focused studies. In Oman, 70% of dementia caregivers reported high burden [25] and in Saudi Arabia, caregivers of Alzheimer’s patients had moderate to high burden [30] with women reporting greater strain.

Our findings show that reporting burden is positively associated with reporting mental disorders, and this is consistent with findings from previous research [7, 8, 11, 27, 31]. Caring for older adults requires physical and emotional involvement which increases the level of stress and physical exhaustion for the caregiver especially if the care-recipient suffers from dementia or other mental health disorders [32].

The wide confidence interval for the association between caregiver burden and caregiver mental health reflect limited precision and likely result from the small proportion of caregivers reporting burden (*n* = 14) and mental health disorders (*n* = 27). Therefore, the effect estimates should be viewed as indicative rather than precise estimates that is directionally consistent with international evidence. Nonetheless, our results highlight the importance of the impact of care-recipient needs on caregiver outcomes; those providing frequent and hands-on care experienced higher burden, similar to prior studies in the region and globally [32]. In our study, we used care arrangement as a proxy for disease severity and our results show significant impact of care arrangement on caregiver burden.

Similar to previous findings, the majority of caregivers were females [7, 32]. This might be explained by the patriarchal culture in our society where caregiving is traditionally seen as a female responsibility. This is also aligned with existing theories in the literature such as Wuest’s feminist caring theory and Social constructivist theory [2, 10]. Notably, all caregivers who felt burden in our sample were females and none were males underscoring gendered caregiving expectations and strain in Lebanese society.

Our findings are similar to the findings in previous research regarding the significant impact of female gender [7], being a co-resident [33] and providing hands-on care [34] on greater caregiver burden.

Unlike findings from previous research [7, 11], we couldn’t detect a modifier effect for gender. This could be explained by the lack of power in our sample, as only 33 caregivers in our sample were male, reducing the ability to detect interaction effects. A larger sample of males is needed to be able to examine the effect modification of gender on the association. This is important to examine, as the proportion of male caregivers is on the rise [35] and recently, there has been an increased interest in the literature on the experience of male caregivers [36, 37]. Our sampling frames cover three governorates and is therefore not nationally representative of Lebanon. Findings should be generalized with caution. A design targeting caregivers as the primary sampling unit across all governorates would better support external validity. Finally, although the ZBI-22 is validated in Arabic, it may not fully capture the stress caregivers feel, where admitting burden can conflict with the cultural and religious expectations of responsibility, duty and honor. Future work should consider culturally adapted burden tools and mixed methods designs to capture lived caregiving experiences more fully.

### Strengths

One of the primary strengths of this study is its contribution to providing new insights on caregiver burden level, caregiver mental health and the impact of caregiver burden on caregiver mental health, which is an under-researched area in Lebanon. The results are valuable to researchers who are conducting relevant studies in countries that share similar sociodemographic, socioeconomic and sociocultural influences. Furthermore, our use of validated and reliable tools with good psychometric properties such as SRQ to assess caregiver mental health and Zarit to assess caregiver burden facilitates comparison of these variables across studies and minimizes information and recall bias where these tools ask about recent and specific experiences; this improves the accuracy of the collected data and enhances internal validity

## Limitations

Our findings are limited by the cross sectional design of the study. Therefore, temporal sequence between burden and mental health can’t be established. Since each older participant identified an informant who would report on his/her cognitive health, this might introduce selection bias as the older participant would most likely refer to a female caregiver. Furthermore, we only included caregivers of older adults who themselves agreed to participate in the original study, which might exclude caregivers who were more stressed or less available. This might introduce further selection bias. In addition, our findings are not conclusive of other confounders mentioned in the literature such as caregiver’s personality traits, emotion regulation, social support, duration since start of caregiving, health status of care recipient, severity of symptoms of care recipient and health status of caregiver [12, 31] which are associated with burden and mental health. Furthermore, our findings don’t take into account the difference in the socio-economic status of the country between 2013 and 2017 and the study is based on data collected prior to the major economic collapse and social instability in Lebanon which may limit its current relevance. The merge of two cross-sectional surveys from two distinct governorates might introduce residual heterogeneity that could reduce internal consistency and increase measurement variance despite standardized training, instruments and protocols. The wide confidence interval around the caregiver burden effect on mental health reflects limited precision due to small subgroup sizes so effect sizes should be interpreted as directional. Cultural norms may have led to under-reporting of burden (particularly among women). Finally, while the study is informative for Lebanon and similar settings, findings may not be generalizable to all Arab or LMIC contexts due to variation in sociocultural norms and caregiving structures.

## Conclusion

This study provides novel insights into the association between caregiver burden and mental health among informal caregivers of older adults in Lebanon. Unlike findings from other regions, the low prevalence of caregiver burden (10%) suggests that cultural norms and lower care intensity shape caregiving experience in Lebanon. Nevertheless, the strong association between burden and mental health underscores a critical yet under-recognized public health concern. Caregivers who felt burdened were 17 times more likely to experience mental health issues, and all burdened caregivers in our sample were women. Our findings highlight the urgent need for culturally sensitive interventions to support female caregivers, particularly those providing hands-on or co-residential care. Community-based programs that offer psychological support, respite services, and targeted training could mitigate burden and improve wellbeing [37]. Given the patriarchal caregiving expectations in Lebanese society, interventions should also challenge gendered caregiving norms and promote shared responsibility within families. Although several interventions have been proposed in the literature [37, 38], it is important to contextualize them to Lebanon before implementing them.

Further studies are warranted to look at burden of care considering a larger number of caregivers to see which type of patients need more attention in order to know who to target. For instance, we need to see how burden gets affected in different kinds of people such as those with dementia, cognitive decline, physical disabilities and neurodevelopmental disorders. Future research should consider using a cohort or longitudinal design to better assess the temporal relationship of burden and mental health. Furthermore, a mixed-methods research is also recommended to gain a more comprehensive understanding of caregiver’s perception of burden and their coping mechanisms – interviews that capture the nuances of cultural expectations and norms.

Overall, this study should be interpreted as an early signal that aligns with global caregiving literature while offering context-specific insights from Lebanon. Larger, longitudinal, and culturally informed studies are needed to deepen understanding of caregiver burden, gender dynamics, and mental health and to guide the development of supportive policies and services.

## Supplementary Information

Supplementary Material 1.

## Data Availability

The dataset analyzed during the current study is publicly available in the Zenodo repository at: 10.5281/zenodo.18370332
