# Supplementary material for: Caregiver burden and mental health among informal caregivers of older adults in Lebanon
Source: Discov Public Health. 2026 Jan 31;23(1):114. doi: 10.1186/s12982-026-01466-4 (PMC12860812; doi:10.1186/s12982-026-01466-4)
Supplement: Supplementary file 1 — Supplementary Material 1. [file 12982_2026_1466_MOESM1_ESM.docx]

**Supplementary Appendix 1 – Sampling Methodology**

**Methods**

## Study population and sample

The first study collected data from 502 older adult participants from Beirut and two districts of Mount Lebanon governorate (Shouf and Aley) using a multi-stage random sampling approach. Referencing a sampling frame used in another study, Beirut was divided into 594 clusters each containing 50 residential buildings and 60 clusters were randomly selected. Since no existing sampling frame was available for Shouf and Aley districts, villages and towns were randomly selected and weighted by size. All households in the selected clusters were approached where individuals aged 65 and older were identified and one eligible participant was randomly selected per household.
